# Physical-chemical influences and cell behavior of natural compounds on titanium dental surfaces

**DOI:** 10.1590/0103-6440202305582

**Published:** 2023-12-22

**Authors:** Patricia Milagros Maquera-Huacho, Gabriel Garcia de Carvalho, Miguel Jafelicci, Elcio Marcantonio, Denise Madalena Palomari Spolidorio

**Affiliations:** 1 Department of Physiology and Pathology, School of Dentistry, São Paulo State University(Unesp), Araraquara, SP, Brazil; 2 Institute of Chemistry, Laboratory of Magnetic Materials and Colloids, São Paulo State University(Unesp), Araraquara, SP, Brazil; 3 Department of Diagnosis and Surgery, School of Dentistry, São Paulo State University(Unesp), Araraquara, São Paulo, Brazil

**Keywords:** Dental Implants, Surface Properties, Chlorhexidine, Natural compounds, Fibroblasts, Osteoblasts, in vitro

## Abstract

The present study evaluated the influence of carvacrol, terpinene-4-ol, and chlorhexidine on the physical-chemical properties of titanium surfaces, cell viability, proliferation, adhesion, and spreading of fibroblasts and osteoblasts in vitro. Titanium surfaces (Ti) were treated with Carvacrol (Cvc), Terpinen-4-ol (T4ol), Chlorhexidine (CHX), DMSO, and ultrapure water (Control group). Physical-chemical modifications were evaluated by surface wettability, the surface free energy (SFE) calculated from the contact angle values using the Owens-Wendt-Rabel-Kaeble (OWRK) equation, scanning electron microscopy (SEM) and energy dispersive spectrometry probe (EDS) system. Cells were seeded onto Ti-treated surfaces and incubated for 24 h and 72 h, then evaluated by Alamar blue assay and fluorescence microscopy. Surfaces treated with Cvc and T4ol showed the presence of Na, O, and Cl. All surfaces showed hydrophilic characteristics and SFE values between 5.5 mN/m and 3.4 mN/m. On the other hand, EDS peaks demonstrated the presence of O and Cl after CHX treatment. A reduction of cell viability and adhesion was noted on titanium surfaces treated with CHX after 24 and 72h. In conclusion, the results indicate that the decontamination with Cvc and T4ol on Ti surfaces does not alter the surface proprieties and allows an adequate interaction with cells involved in the re-osseointegration process such as fibroblasts and osteoblasts.

## Resumo

O presente estudo avaliou a influência do carvacrol, terpineno-4-ol e clorexidina nas propriedades físico-químicas de superfícies de titânio, viabilidade celular, proliferação, adesão e esplhamento de fibroblastos e osteoblastos in vitro. Superfícies de titânio (Ti) foram tratadas com Carvacrol (Cvc), Terpinen-4-ol (T4ol), Clorexidina (CHX), DMSO e água ultrapura (Grupo Controle). As modificações físico-químicas foram avaliadas pela molhabilidade da superfície, a energia livre de superfície (ELS) calculada a partir dos valores do ângulo de contato usando a equação de Owens-Wendt-Rabel-Kaeble (OWRK), microscopia eletrônica de varredura (MEV) e espectroscopia de raios X por energia dispersiva (EDS). As células foram semeadas em superfícies tratadas com Ti e incubadas por 24 h e 72 h, e avaliadas pelo ensaio Alamar blue e microscopia de fluorescência. As superfícies tratadas com Cvc e T4ol mostraram a presença de Na, O e Cl. Todas as superfícies apresentaram características hidrofílicas e valores de ELS entre 5,5 mN/m e 3,4 mN/m. Por outro lado, os picos de EDS demonstraram a presença de O e Cl após o tratamento com CHX. Uma redução da viabilidade celular e adesão foi observada em superfícies de titânio tratadas com CHX após 24 e 72h. Em conclusão, os resultados indicam que a descontaminação com Cvc e T4ol em superfícies de Ti não altera as propriedades da superfície e permite uma interação adequada com células envolvidas no processo de reosseointegração como fibroblastos e osteoblastos.

## Introduction

To date, numerous strategies have been suggested for the treatment of peri-implant disease, mechanical instrumentation, chemical decontamination, use of lasers, implantoplasty, and electrolysis, that is just to name a few. None of the mentioned strategies are superior to the others ^1^. However, considering the highly demanding challenge and the infectious nature of peri-implantitis, the combination of strategies appears to be the best measure to achieve biofilm disruption and disease resolution ^2^,^3^. Based on this, chlorhexidine is commonly used as an adjuvant in surgical and non-surgical therapies due to its substantivity ^4^, biological properties, and broad-spectrum antibacterial activity ^5^.

Moreover, researchers are interested in the investigation of alternative therapies using herbal derivatives for the control of microorganisms; these are expected to minimize potential side effects, such as bacterial resistance and surrounding tissue damage. Terpinen-4-ol (T4ol) is the main component of the essential oil Melaleuca alternifolia and has been highlighted mainly because of its antimicrobial and anti-inflammatory activity ^6^ ^), (^ ^7^ ^)(^ ^8^. Carvacrol (Cvc) is the main natural constituent of Thymus vulgaris, Carum copticum, and Oreganum spp. (volatile oils) ^9^, presents different properties, such as, antimicrobial, antioxidant, antimutagenic, antigenotoxic, and hepatoprotective, emphasizing its wide spectrum antimicrobial activity on pathogenic bacteria and yeasts including drug-resistant biofilm ^6^,^10^.

The impact of treatment modalities on the surface topography and physical-chemical properties of implants is considered a key aspect, and it may impair the re-osseointegration. The use of cold atmospheric pressure argon plasma did not enhance microorganism elimination and osteoblast spreading, but erythritol powder appears to be effective ^11^. Sulfonic/sulfuric acid solution coupled with erythritol, amorphous silica, and 0.3% chlorhexidine showed a significant decontaminant effect and a higher percentage of cell proliferation compared to other solutions tested ^12^. Acidic environments can introduce noticeable morphological changes and corrosion on the surface of titanium implants ^13^. In this way, various antimicrobial agents and methods have been suggested for implant surface decontamination to achieve re-osseointegration. Some studies investigated the biocompatibility of the implant surfaces with the cells of peri-implant tissues after different treatments ^14^-^16^. Moreover, there is still a lack of information to get a general understanding of the influence of natural compounds on titanium (Ti) surface properties as a chemical structure and mainly on cell adhesion and proliferation around dental implants.

There is limited information to support those titanium surface alterations induced by decontamination interventions lead to compromised biological response during the healing phase ^11^-^16^. This study was conducted to evaluate the influence of chlorhexidine and natural compounds such as Carvacrol and Terpine-4-ol on the physical-chemical properties of titanium surfaces. Additionally, evaluation of the effect of these surface modifications on cell viability, proliferation, adhesion, and spreading of Fibroblasts L929 and Osteoblasts SaOs-2 was performed.

## Material and methods

### Test solutions and specimen treatment

Carvacrol and Terpinen-4-ol (Sigma-Aldrich, USA) stock solutions were solubilized in Dimethyl sulfoxide (DMSO) 0.4% and 2% (Sigma-Aldrich, USA) ^17^,^18^. In the current study, Cvc and T4ol were prepared at 0.5% and 0.059%, respectively due to their different antimicrobial and anti-inflammatory properties focused on the treatment of periodontal disease ^10^,^18^,^8^. Chlorhexidine 0.2% was used as a positive control antimicrobial agent ^19^.

Sterile titanium discs with SLA surface produced by using TiO_2_ microparticles for blasting and subsequent acid conditioning (5 mm; thickness 2 mm), kindly provided by *Implacil De Bortoli* (Brazil), were used. The specimens were treated with Cvc 0.5%, T4ol 0.059%, DMSO 2%, and CHX 0.2% according to clinical protocols; the durations of treatment were 5 minutes ^20^,^21^. Ultrapure water was used as a negative control group. Subsequently, the samples were dried at room temperature ^21^.

### Surface wettability and surface-free energy

The surface wettability and surface free energy (SFE) was calculated from the contact angle (n = 9) using the sessile drop technique (Young-Laplace equation) as described previously ^22^. Liquids with different polarities: distilled water (52).^2^, ethylene glycol (19.0), polyethylene glycol (13.6), and diiodomethane (2.6) were dropped (0.08 µL) onto the discs. After, the contact angle had been measured through a goniometer; the arithmetic mean and standard deviation were calculated for the SFE of each group, using the Owens-Wendt-Rabel-Kaeble (OWRK) equation ^23^.

### Scanning electron microscopy and energy dispersive spectrometry

Three samples were analyzed using scanning electron microscopy (SEM) and energy dispersive spectrometry (EDS) (JEOL JSM-7500F, JEOL Ltda, USA) to investigate surface topography and the chemical elements present after decontamination with Cvc, T4ol, DMSO, and CHX.

### Cell growth and proliferation

Mouse fibroblast L929 and Human osteoblast-like SaOs-2 cells were cultured in Dulbecco’s modified Eagle’s medium (DMEM; Sigma-Aldrich, USA) supplemented with 10% fetal bovine serum (FBS; Gibco, USA), 100 IU/mL penicillin, 100 μg/mL streptomycin and 2 mmol/L glutamine (Gibco, USA) in a humidified incubator with 5% CO_2_and 95% air at 37°C. The cells were sub-cultured until an adequate number of cells were obtained for the study.

Carefully, the discs were placed in sterile 96-well plates and 5x10^4^ cells/well (200 μL) were placed onto the surfaces and maintained in the incubator under the described conditions for 24 h and 72 h. Then, after each period the cells were evaluated using Alamar Blue® (Invitrogen, USA) assay (n=9) and fluorescence microscope (n=3).

Alamar Blue® assay was performed to assess the metabolic activity by the fluorimetric indicator and cell proliferation after each period. Alamar Blue® is based on the incorporation of the oxidation indicator that shows the color change in response to chemical reduction of the culture medium due to aerobic respiration. Thus, Alamar Blue dye (10%) prepared in cell culture medium without FBS was added to the cells and incubated at 37 °C in 5% CO_2_ for 4 h. After this period, an aliquot of each well (100 μL) was transferred to a new 96-well plate and the fluorescence of the samples was read in a spectrophotometer (Synergy - H1 - Biotek Winooski, USA) at the wavelength of 570 nm (reduction) and 600 nm (oxidation).

### Analysis by fluorescence microscope

After 24 h and 72 h of cell culture, Actin Red 555 ReadyProbes® (Invitrogen, USA), which is a selective high-affinity reactant of actin filaments where the actin cytoskeleton is stained with TRITC-phalloidin (red) and the nucleus with Hoechst 33242 (blue) evaluated adhesion and spreading of fibroblasts and osteoblasts on disc surfaces. Briefly, samples were fixed with paraformaldehyde for 15 minutes and washed with phosphate buffer solution (PBS). Then, Triton x-100 (0.1%) was added for 10 minutes and washed with PBS. The cells with actin marker were incubated for 30 minutes, the reagent was aspirated, and Hoechst was added for 10 minutes. Finally, the cells were analyzed using a fluorescence microscope EVOS FL Imaging System (Thermo Fisher Scientific, USA) with red and blue filters.

### Statistical analysis

Statistical analysis was performed using SPSS software (USA). Contact angle values ​​were submitted to Kruskal-Wallis and Mann-Whitney nonparametric tests for comparison of paired groups since they did not present normal distribution. All the absorbance data of Alamar Blue analysis were compared using two-way ANOVA with a significance level of 5 % (*p* < 0.05).

## Results

### Surface Wettability and SFE

The contact angle and surface-free energy are shown in **Table 1**. Surface wettability (using distilled water) showed that all Ti surface treatments (DMSO, CHX, T4ol, and Cvc) led to a decrease in the contact angles when compared to the control group: 67.8±1.9 (*p* < 0.05).

After measuring the contact angles and determining the wettability on the tested surfaces, the surface free energy was calculated for each group about the polarity of the tested solution (distilled water, ethylene glycol, polyethylene glycol, and diiodomethane) by applying the OWRK equation ^23^. According to the values obtained and corresponding to the polar capacity of the surfaces, the control group presented a higher SFE value: of 5.5 mN/m, and the surfaces decontaminated with DMSO, CHX, T4ol, and Cvc presented lower ELS values: of 3.4 mN/m, 3.7 mN/m, 3.8 mN/m and 4.0 mN/m respectively.

**Table 1:** Values of Surface-free Energy (SFE) (mN/m), mean, and standard deviation of the contact angles in the different groups evaluated.

Groups	Control	DMSO 2%	CHX 0.2%	T4ol 0.059%	Carvacrol 0.5%
Contact angle (*degree*)					
Distilled water	67.8±1.9	18.21±1.6*	31.1±0.8*	25.73±0.6*	33.9±1.3*
Ethylene glycol	28.3±0.4	20.7±0.5	21.4±0.7	18.7±0.8	23.7±0.7
Polyethylene glycol	24.9±2.8	30.0±0.8	25.5±0.8	24.6±0.7	22.6±0.9
Diiodomethane	41.2±1.6	26.2±0.9	42.0±1.3	28.0±0.9	25.6±1.3
SFE (mN/m)	5.5	3.4	3.7	3.8	4.0

*Significant difference (*p*<0.05) compared with the control group.

*Note*. The surface-free energy (mN/m) is shown as the value of its polar component.

### Scanning electron microscopy and energy dispersive spectrometry analysis

**Figure 1:**
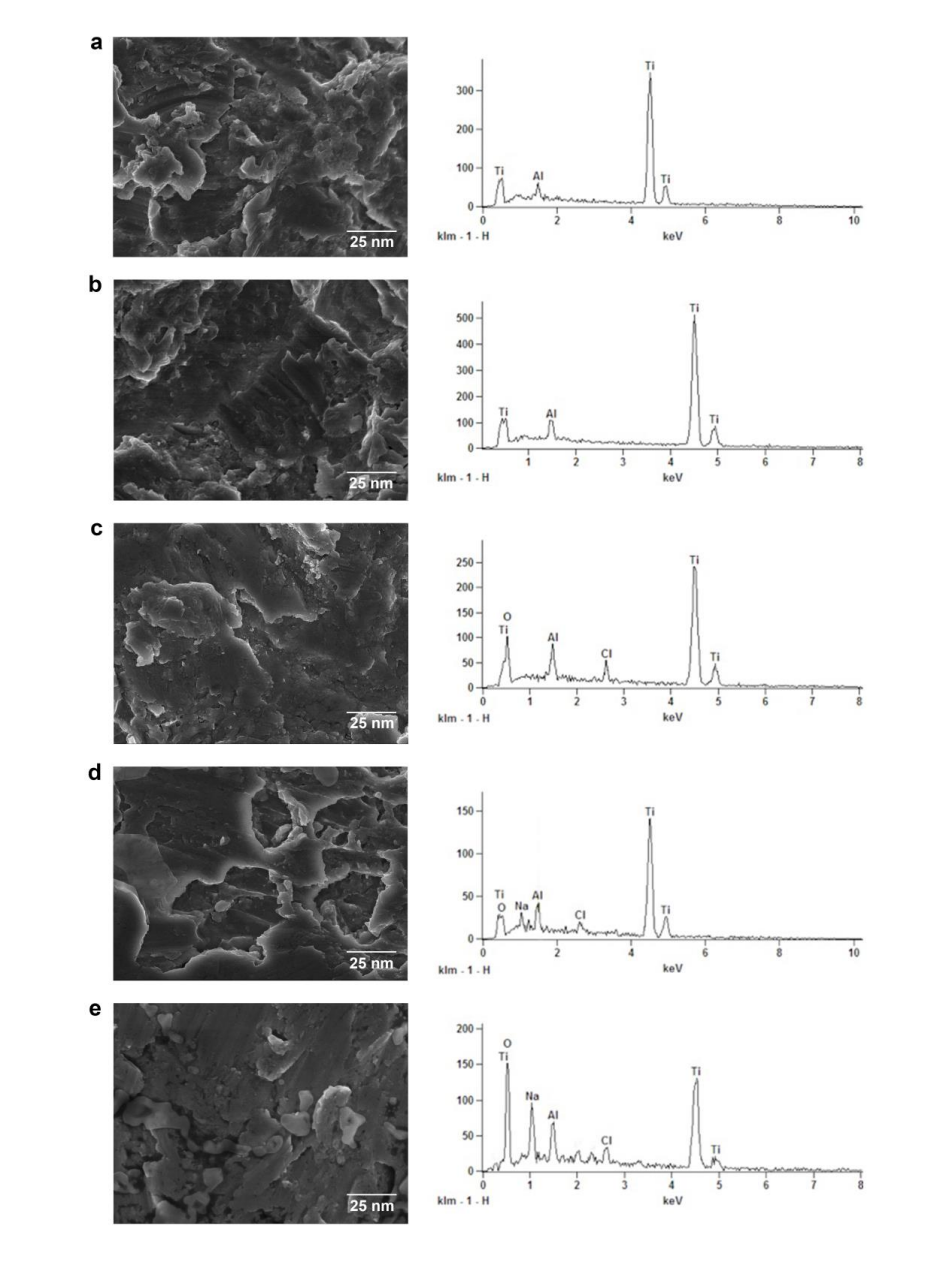
Scanning electron microscopy (SEM) images and energy dispersive spectroscopy (EDS) of each group: Control group (a), DMSO 2% (b), CHX 0.2% (c), T4ol 0.059% (d) and Carvacrol 0.5% (e).

Specimens from all groups were analyzed descriptively based on SEM findings and, quantitatively and qualitatively on EDS, results can be seen in Figure 1. The images of the control group and DMSO revealed the presence of chemical elements: Titanium (Ti) and Aluminum (Al). For the CHX group, the following elements were identified: Oxygen (O), Titanium (Ti), Aluminum (Al), and Chlorine (Cl). Also, for T4ol and Cvc groups: Oxygen (O), Titanium (Ti), Sodium (Na) Aluminum (Al), and Chlorine (Cl) were mainly observed.

### Cell viability and cell proliferation

**Figure 2:**
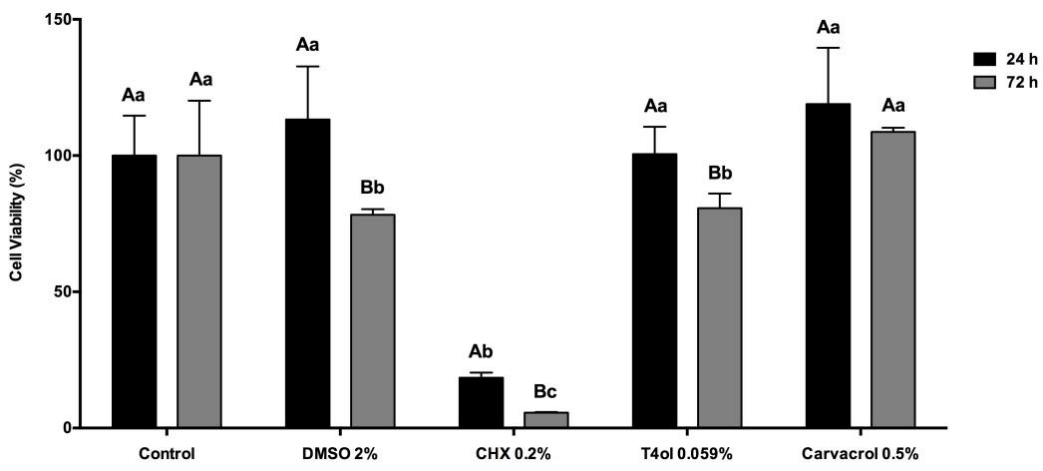
Cell viability of Fibroblast L929 (%) for each experimental group at the set evaluated period. Uppercase letters allow comparison among the evaluated periods for each experimental group. Lowercase letters allow comparison among the groups within each evaluated period. Different letters show significant differences (ANOVA two-way, p < 0,05).

The results showed that the viability of fibroblast L929 (Figure 2) was affected when cultivated on surfaces treated with DMSO, CHX, and T4ol at 72 h (*p* < 0,0001). A greater decrease in cell viability was observed on surfaces treated with CHX at 24 h and 72 h. However, the control group as well as those treated with Cvc did not affect cell viability during the evaluated periods (*p* > 0.05).

**Figure 3:**
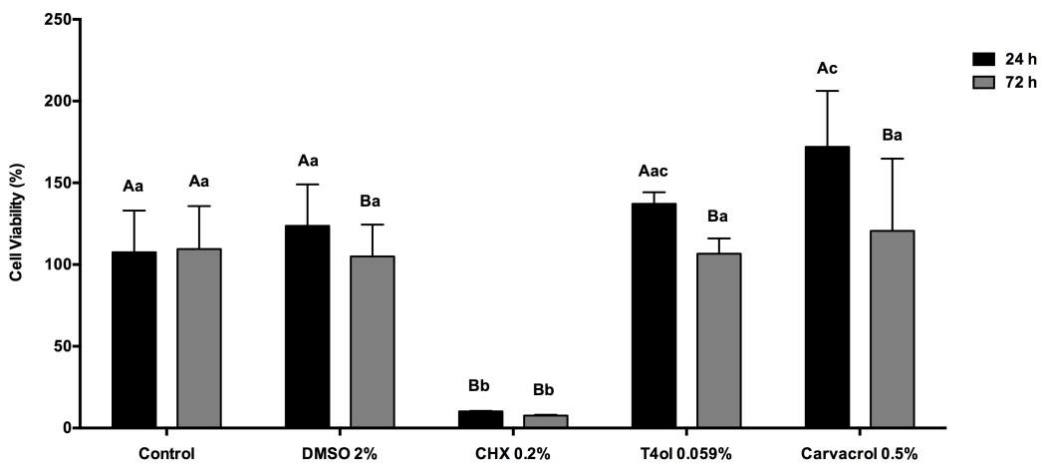
Cell viability of osteoblasts SaOs-2 (%) for each experimental group at the set evaluated period. Uppercase letters allow comparison among the evaluated periods for each experimental group. Lowercase letters allow comparison among the groups within each evaluated period. Different letters show significant differences (ANOVA two-way, *p* < 0,05).

The results of osteoblasts SaOs-2 viability are shown in Figure 3. Surfaces treated with DMSO and Cvc show decreased of cell viability after 72 h (*p* < 0.0001). However, surfaces treated with CHX revealed a significant decrease at 24 and 72 hours (*p* < 0.0001).

**Figure 4:**
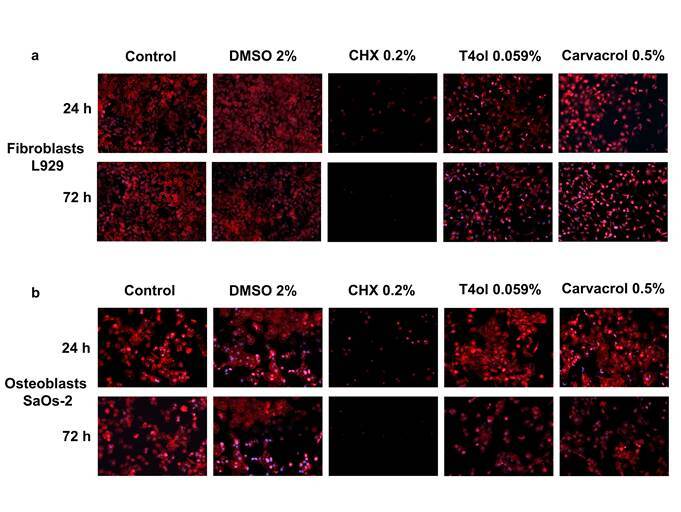
Photomicrographs of cytoskeletal identification of actin (red) of fibroblasts L929 (a) and osteoblasts SaOs-2 (b) adhered to treated surfaces for 24 and 72 h. Nuclei were stained with Hoescht (x10).

The adhesion and spreading of fibroblasts L929 and Osteoblasts SaOs-2 (Figure 4) were assessed by the immune-identification of actin filaments. For both cell lines, the photomicrographs showed extensive cell damage in the Ti surfaces treated with CHX.

## Discussion

The main goal of peri-implantitis treatment is achieving the infection resolution and the return of tissue health patterns, after that an environment conducive to re-osseointegration is desirable. Taking this into account, the evaluation of dental implant surface modifications after decontamination with different therapies becomes important knowledge for the selection of the best biocompatible treatment of periimplantitis. Therefore, the present study, it was evaluated surface modifications of titanium surfaces after treatment with CVC and T4ol, and the biocompatibility of these surfaces as well as the cellular behavior of fibroblasts and osteoblasts.

The decontaminated surface must have characteristics suitable for cell adhesion and proliferation. In this context, cell adhesion can be affected by different surface properties such as surface energy, roughness, and chemical composition ^24^ ^) , (^ ^25^. Within these properties, the wettability is also an important parameter to understanding the cell behavior. Contact angle <90º tends to wet more the surface and it becomes more interactive and hydrophilic ^26^. In this study, the Ti surfaces exposed to all solutions tested have shown a hydrophilic characteristic, and significant reductions in the contact angle compared with the control group (67.8±1.9) were observed for all solutions tested, which demonstrates increased wettability (Table 1). In the same way, Al-Radha et al. ^27^, reported similar wettability data on titanium discs treated with cinnamon oil, clove oil, and CHX, corroborating our results. Overall, decontaminated surfaces showed higher wettability than control surfaces in another similar study ^28^. In contrast, other study revealed the hydrophobic characteristic after decontamination of the implant ^29^. Although hydrophilic surfaces can promote an environment conducive to osseointegration by improving osteoblast maturation ^30^,^31^. According to Wennerberg et al., low concentrations of carbon were observed in hydrophilic surfaces, while hydrophobic surfaces displayed higher amounts ^32^. Significant amounts of carbon were not observed after EDS assay in our study.

The SFE represents the surface tension of each material and it is associated with the state of equilibrium of the atoms present in the outer layer. Then, if the SFE is considered high, there are more activated ions available on this surface and it can interact more efficiently with surrounding tissues, then cell adhesion is improved ^33^. The current results of SFE are presented about the polar component and the surfaces treated with natural compounds (T4ol and Cvc), SFE values of 3.8 and 4.0 mN/m were observed, respectively. No significant difference was identified in SFE when compared to the control group.

All known in the process of osseointegration is that after the first contact between blood and titanium, the gradual inclusion of Ca and P ions into the Ti oxide layer occurs (TiO) ^34^. Later, a layer of fibroblast-like cells elongates on the implant surface ^35^. After 1 week of the first contact, a thin layer of an osteoid matrix still in the process of calcification is seen, the osteoblasts act as a center of ossification in this provisional matrix, and it is remodeled into lamellar bone further ^36^. In this context, our results showed the presence of TiO in greater amounts on surfaces treated with Cvc. Other studies in which the effects of decontamination solutions on the surface of titanium were tested, found the presence of titanium, oxygen, aluminum, and carbon as the main components of all the treated titanium disks ^29^, ^13^.

The surfaces treated with CHX presented the lowest level of cell viability for both types of cells tested, which can be attributed to the presence of chlorine found on its surface on EDS. We speculate that when the discs treated with CHX come into contact with the cells in an aqueous medium, a dissociation of the CHX molecule occurs, releasing hydrogen and chlorine ions to the medium, allowing their ionic interaction and turning the medium into an acidified environment, and consequently damaging the cell viability. However, more research is needed in this area.

Acidic environments can introduce noticeable morphological changes and corrosion on the surface of titanium implants ^13^. These results differ from those reported by Ungvári et al. ^21^, who found the presence of carbon and oxygen on surfaces treated with CHX gel. On the other hand, surfaces treated with Cvc and T4ol showed the presence of sodium and chlorine ions, when bound to each other, NaCl (salt) is expected to be comprised. This salt might not have a significant impact on altering pH and that is probably why cell adhesion of fibroblast L929 and Osteoblasts SaOs-2 can be seen in Figure 4. However, some difference in the appearance of the Saos 2 cells after 24 h of titanium surface decontamination has already been reported before ^29^.

Although the use of CHX has not been shown to modify significantly the surface properties in terms of topography and wettability, the cell viability results have shown a significant decrease after CHX treatment in both cell types at 24 h and 72 h, and impacts on actin cytoskeleton can also be seen in the fluorescence images of this group. This effect on the actin cytoskeleton reduces the adhesion ability of the cells affecting matrix components and receptors ^16^, ^37^, ^38^. CHX’s cytotoxicity against osteoblasts has already been elucidated by other research groups ^39^,^40^ Similar results of cell viability were described by Chellini et al. ^16^, using osteoblast-like Saos2 on surfaces pre-treated with CHX 0.2% for 5 minutes. The cationic nature of the CHX molecule permits the interaction with implant and biofilm when it is slowly released over 24 h (CHX 0.2%); this effect is responsible for the substantivity characteristic ^41^. This property might improve its antibacterial effect and at the same time influence the cell viability of fibroblasts and osteoblasts once it is not easily eliminated ^28^. It is also known that CHX has toxic effects on different cell types, causing total cell death by adenosine triphosphate (ATP) depletion and it can induce the inhibition of mitochondrial activity, protein and DNA synthesis, and cell proliferation ^42^.

It is important to highlight the cell behavior observed after the treatment of Ti surfaces with the natural compounds. It is noted that both, T4ol and Cvc were not able to disturb the cell function of fibroblasts and osteoblasts, which is confirmed by the cell viability, proliferation, and morphology aspect in the fluorescence microscopy. No difference in cell viability was observed when fibroblast L929 was grown on surfaces treated with T4ol and Cvc after 24h. After 72h, a cell viability reduction was observed in the T4ol group which might be explained due to the use of DMSO as a diluent, since the same result was observed in the DMSO group. For human osteoblasts like SaOs-2, there was an increase in cell viability after the treatment of the Ti surfaces with natural compounds, mainly in the CVC group where this increase is considered significant when compared to the control group at 24h. After 72h some reduction was noted, which is probably due to the use of DMSO again. In a recent study, more than 50% of the Ti decontaminated surface was covered by adherent MG-63 cells after treatment with erythritol, amorphous silica, 0.3% chlorhexidine, and a sulfonic/sulfuric acid solution while the other treatment measures reached less than 33% ^12^. The results showed in the present study are incipient to fully investigate the cell behavior involved in the re-osseointegration process. Osteoblast differentiation and the expression of bone markers, as well as bone formation (three-dimensionally), should be explored in further investigations. Additionally, in a clinical setting there are different kinds of cells around the implant and the complex multispecies biofilm may play an additional challenge, these are some of the limitations of the present study.

## Conclusion

 Based on the findings of the present study, it was demonstrated that titanium surfaces treated with natural compounds such as Carvacrol and Terpine-4-ol allow the proliferation, adhesion, and spreading of fibroblasts and osteoblasts, cells involved in the re-osseointegration process. In contrast, Ti surfaces exposed to Chlorhexidine showed a relevant level of cytotoxicity which may disturb the re-osseointegration process. The physical-chemical properties and topography of titanium surfaces were not impacted by the treatments investigated in this study.
